# Modulatory Effect of Gliadin Peptide 10-mer on Epithelial Intestinal CACO-2 Cell Inflammatory Response

**DOI:** 10.1371/journal.pone.0066561

**Published:** 2013-06-18

**Authors:** Antonella Capozzi, Olimpia Vincentini, Pietro Gizzi, Alessandra Porzia, Agostina Longo, Cristina Felli, Vincenzo Mattei, Fabrizio Mainiero, Marco Silano, Maurizio Sorice, Roberta Misasi

**Affiliations:** 1 Department of Experimental Medicine, Sapienza University, Rome, Italy; 2 Unit of Human Nutrition and Health, Istituto Superiore di Sanità, Rome, Italy; 3 Experimental Medicine and Environmental Pathology Laboratory, “Sapienza” University, Rieti, Italy; Duke University Medical Center, United States of America

## Abstract

Celiac Disease (CD) is a chronic inflammatory enteropathy, triggered in genetically susceptible individuals by dietary gluten. Gluten is able to elicit proliferation of specific T cells and secretion of inflammatory cytokines in the small intestine. In this study we investigated the possibility that p10-mer, a decapeptide from durum wheat (QQPQDAVQPF), which was previously shown to prevent the activation of celiac peripheral lymphocytes, may exert an inhibitory effect on peptic-tryptic digested gliadin (PT-Gly)-stimulated intestinal carcinoma CACO-2 cells. In these cells, incubated with PT-Gly or p31-43 α-gliadin derived peptide in the presence or in the absence of p10-mer, IRAK1 activation and NF-kB, ERK1/2 and p38 MAPK phosphorylation were measured by immunoblotting, Cyclooxigenase 2 (COX-2) activity by PGE-2 release assay, and production of cytokines in the cell supernatants by ELISA. Our results showed that pre-treatment of CACO-2 cells with p10-mer significantly inhibited IRAK1 activation and NF-kB, ERK1/2 and p38 MAPK phosphorylation, as well as COX-2 activity (i.e. PGE-2 release) and production of the IL-6 and IL-8 pro-inflammatory cytokines, induced by gliadin peptides. These findings demonstrate the inhibitory effect of the p10-mer peptide on inflammatory response in CACO-2 cells. The results of the present study show that this p10-mer peptide can modulate "in vitro" the inflammatory response induced by gliadin peptides, allowing to move towards new therapeutic strategies. Turning off the inflammatory response, may in fact represent a key target in the immunotherapy of celiac disease.

## Introduction

Celiac disease (CD) is a chronic inflammatory enteropathy with an autoimmune pathogenesis, triggered in genetically susceptible individuals by dietary gluten, the alcohol protein fraction of cereals, such as wheat, rye and barley. Gliadin is the main protein from wheat gluten. CD is histologically characterized by villous atrophy, crypt cell hyperplasia and increased number of intraepithelial lymphocytes. In addition, patients with celiac disease typically develop antibodies to gliadin and autoantibodies specific for endogenous enzyme transglutaminase 2 (TG2) [1]. At present, many gluten peptides have been described to elicit proliferation and secretion of cytokines, such as interferon-γ, of gluten specific T cells in the intestinal mucosa of CD patients [2], [3]. Susceptibility to celiac disease is strongly associated with major histocompatibility complex (MHC) class II molecules, HLA-DQ2 and HLA-DQ8. In particular, the immune response, directed against specific gluten antigens, leads to destruction of intestinal epithelial cells (IECs).

Although gluten is known to play a role in activating gluten-specific T cells in the lamina propria, recent evidences support the notion that gluten affects the innate immune response [4]–[6]. Thus, both innate and adaptive immune responses are necessary to trigger the gluten-dependent mucosal inflammation [7]. Indeed, the α-gliadin-derived peptide p31–43 is able to activate the innate immune response in celiac mucosa. Moreover, the activation of the mucosal innate immunity by p31–43 has been demonstrated to be required to trigger the immunogenicity of p33-mer, another α-gliadin derived peptide [8]–[10].

Activation of intestinal epithelial cells plays a pivotal role in CD pathogenesis [8] mediating gut inflammation. Intestinal epithelial cells represent an important component of innate immunity and activate sophisticated responses to inflammatory stimuli. The manner in which intestinal epithelial cell polarity affects responses to inflammatory stimuli is largely unknown, but it is clear that intestinal epithelial cells actively participate in directing an inflammatory response [11]. Although the vast majority of bacterial and food components do not elicit intestinal inflammation, it is known that pathogens that cause acute inflammation do activate the NF-kB pathway, resulting in a regulation of genes encoding proinflammatory cytokines, chemokines and adhesion molecules [11], [12].

At present, the only known treatment of CD is the life-long withdrawl of gluten containing food from the diet.

We described p10-mer, a decapeptide (sequence QQPQDAVQPF) [13] that prevents the activation of peripheral celiac lymphocytes by gliadin peptides [14] and promotes a shift from a Th1-type response toward a Th2-type response in celiac disease [15]. In the present study we evaluated "in vitro" the modulating effect of p10-mer on gliadin-triggered intestinal epithelial cell inflammation. Our results indicated first that this peptide is able to prevent IRAK1 and NF-kB activation, induced by gliadin peptides, in intestinal CACO-2 cells.

## Materials and Methods

### Cell culture

CACO-2/TC7 cells [16] were cultured in Dulbecco's Modified Medium (high glucose) supplemented with 1% non–essential amino acids and containing 10% fetal calf serum (FCS), 2 mM L-glutamine, 100 U/ml penicillin, 100 mg/ml streptomycin, and 1 mM Hepes (Invitrogen, Carlsbad, CA, USA) at 37°C in a humified 5% CO_2_ atmosphere. The culture medium was replaced three times a week. Subculture was performed at 80% of confluence. All experiments were performed between passages 65 and 78.

### Gliadin preparation and peptic-triptic digestion

The peptic-tryptic digest of gliadin (PT-Gly) is a useful tool to study the biological effects of gliadin. The PT-Gly contains some gliadin peptides that show different toxic activity in CD patients tissues. The alcohol-soluble protein fraction from whole cereal flour of bread wheat (*Triticum aestivum*, variety S Pastore) was extracted and subjected to peptic-tryptic digestion, as previously described [14], [17]. Briefly, gliadin was separated from wheat flour by a 70% ethanol aqueous solution. Then, gliadin was exposed at 37°C to pepsin (Sigma Chem Co, St. Louis, MO USA), for 2 hours pH 2.0 and, then, to trypsin (Sigma) for 4 hours pH 8.0. Afterwards, the incubation was stopped adjusting pH to 7.0 and the samples were lyophilized and stored at −20°C until used. Gliadin preparation was assayed for endotoxin by using a reagent kit (QCL-100; BioWhittaker, Walkersville, MD USA); they were found to have endotoxin concentrations <0.5 enzymatic U/ml.

### Peptide preparation

The sequence p10-mer (QQPQDAVQPF; MW = 1157 D) and biotinylated gliadin peptide p31–43 (LGQQQPFPPQQPYPQPQPF) were synthesized (Primm Company, Milano, Italy) by the solid-phase method (model 431A; Applied Biosystems, Foster City, CA, USA) and purified up to 99% with the use of reverse phase HPLC (5020 system; Varian Inc, Walnut Creek, CA, USA). Peptides were used in the experiments at a concentration of 50 µg/ml.

### Preparation of cell extracts

CACO-2 cells were pre-treated for 1 hour with p10-mer (50 µg/ml) and then stimulated with PT-Gly (1 mg/ml) or 31–43 α-gliadin peptide (p31–43) (50 µg/ml), without removing pre-treatment, at various times (10 min-3 hours) at 37°C, in 5% CO_2_. After treatment, cells were placed on ice, washed once in Phosphate Buffered Saline (PBS) and scraped in PBS. For the preparation of whole cell extracts, cells were resuspended in lysis buffer (20 mM HEPES, pH 7.2, 1% Nonidet P-40, 10% glycerol, 50 mM NaF, 1 mM Na_3_VO_4_, including protease inhibitors, Sigma). DNA was sheared by brief sonication and soluble proteins were recovered after centrifugation of lysates at 15,000× *g* for 15 min at 4°C. Protein concentration was determined by Bradford assay using BSA as a standard (Bio-Rad Lab., Munchen, Germany) and samples were frozen at −80°C.

### Western blot analysis

Equal amounts of whole cellular extract (from unstimulated or stimulated CACO-2/TC7 cells with PT-Gly, p10-mer, PT-Gly+p10-mer, p10-mer+PT-Gly, p31−43, p10−mer+p31–43) were separated in 7.5% SDS-PAGE under reducing conditions and electrophoresed by the use of mini Protein II Dual Slab Cell. The proteins were electrophoretically transferred to PVDF membrane (Bio-Rad Lab.) and then, after blocking with PBS, containing 1% albumin, probed with polyclonal anti-phospho-IRAK1 (Cell Signalling, Inc, Danvers, MA, USA), polyclonal anti-phospho-NF-kB p65 (Cell Signalling, Inc), polyclonal anti-phospho-ERK1/2 (Cell Signalling, Inc), polyclonal anti-phospho-p38 (Cell Signalling, Inc) or monoclonal anti-cyclooxigenase 2 (COX-2) antibodies (Abcam, Cambridge, UK). Bound antibodies were visualized with HRP-conjugated anti-rabbit IgG or anti-mouse IgG (Sigma) and immunoreactivity was assessed by the chemiluminescence reaction using the ECL Western blot system (Amersham Pharmacia Biotech, Little Chalfont, Buckinghamshire, UK). As a control for nonspecific reactivity, parallel SDS-PAGE gels were blotted as described, using an anti-rabbit IgG or anti-mouse IgG (Sigma). As a control for loading of preparation, IRAK1, phospho-NF-kB p65, phospho-ERK1/2, phospho-p38 and COX-2 blotted membranes were stripped and reprobed with monoclonal anti-α-tubulin (Sigma) and with polyclonal anti-IRAK1 (Cell Signalling, Inc), anti-NF-kB p65 (Cell Signalling, Inc), anti-ERK1/2 (Cell Signalling, Inc) or anti-p38 (Cell Signalling, Inc) antibodies, respectively.

### Analysis of Cyclooxigenase 2 activity by PGE-2 production

Briefly, CACO-2/TC7 cells were seeded in 6 wells plates at a density of 500×10^3^ cells and cultured for five days. Cells were then incubated in medium containing 0.5% FBS in the presence or in the absence of p10-mer peptide, as pre-treatment for 1 hour and then stimulated with PT-Gly for 24 hours at 37°C. At the end of the treatment, the culture medium was collected and centrifuged to remove particulates, then stored at −80°C. The PGE-2 concentration released in the sample was quantified using a PGE-2 assay kit (Cayman, Ann Arbor, MI, USA) according to manufacturer's protocol. Supernatants were diluted to a 5-fold dilution to ensure the concentrations of PGE-2 present in the samples were within the linear range of the standard curve for the assay. The PGE-2 concentration of each sample was determined according to the standard curve.

### Cytokine determination

Cells were stimulated as described above for 24 hours at 37°C. After treatment, cell supernatants were collected and frozen at −80°C until analysis. Production of the pro-inflammatory cytokines IL-6 and IL-8 was measured in the cell supernatants using an enzyme-linked immunosorbent assay (ELISA) (Bender MedSystems, San Diego, CA, USA) and quantified using a reference standard curve provided with the kit. The experiments were performed in triplicate for three times.

Cellular supernatants from unstimulated or stimulated CACO-2/TC7 cells with PT-Gly, p10-mer or p10-mer+PT-Gly were tested for IL-17 concentration by enzyme-linked immunosorbent assay (ELISA), using a commercially available ELISA kit (R&D Systems, Inc., Minneapolis, MN, USA), according to the manufacturer's instruction. Simultaneously, unstimulated or stimulated CACO-2/TC7 cells with PT-Gly, p10-mer or PT-Gly+p10-mer were washed with phosphate-buffered saline, fixed with 2% paraformaldehyde for 20 min, permeabilized with 0.5% saponin/1% fetal calf serum, and stained with anti-IL-17A PE (Phycoeritrine) mAb for 30 min at 4°C. Control cell samples were stained with IgG1 PE. Both mAbs were obtained from Biosciences (San Diego, CA, USA). Cells then were analyzed with a FACScalibur (BD Biosciences, San Josè, California, USA), using FlowJo (Treestar, Ashland, OR, USA) software. The percentage of IL-17 producing cells was obtained by setting the lower limit on the basis of a matched isotype IgG1 PE control mAb-stained sample, whose positivity never exceeded 0.5% of gated events.

### Immunofluorescence and flow cytometry analysis of p31-43 expression

CACO-2 cells were grown to 60–70% confluence, seeded on µ-Slide 8 well (Ibidi, Martinsried, Germany) and used for an indirect immunofluorescence assay. Confluent cells were pre-treated for 1 hour with p10-mer (50 µg/ml) and then incubated for 10 min with biotinylated p31–43 (50 µg/ml). Cells were then washed with PBS and fixed with paraformaldehyde 4% in PBS for 30 minutes at room temperature, permeabilized with 0.05% Triton-x100 in PBS for 5 min at room temperature and stained with streptavidin-Alexafluor 488 (1∶400) (Life Technologies, Paisley, UK). The cells were washed for 3 times with PBS and slides were mounted in glycerol/Tris-HCl pH 9.2 (3∶1) and observed at the microscope. The images were acquired using an Olympus U RFL microscope (Olympus, Hamburg, Germany). The images were collected at 512×512 pixels, processed and filtered to minimize background noise.

For cytofluorimetric analysis cells were seeded at a density of 5×10^5^ on 6 wells plates, and pre-treated at confluence with p10-mer (50 µg/ml) for 1 hour and then with biotinylated p31–43 (50 µg/ml) for 10 min. At the end of the treatments cells were detached by the use of trypsin and washed with PBS, fixed with paraformaldehyde 4% in PBS for 30 min at room temperature and permeabilized with 0.05% Triton-x100. Cells were incubated with streptavidin-Alexafluor 488 (1∶400), washed with PBS and analysed by a BD FACSCalibur flow cytometer (Becton Dickinson Bioscences), using FlowJo (Treestar) software.

### Statistics

All the statistical procedures were performed by SAS/STAT Software provided by SAS Institute Inc. (Cary, North Carolina, USA). P values have been calculated with the T-Student test and P values less than 0.05 were considered as significant.

## Results

### Inhibitory effect of gliadin peptide 10-mer on PT-Gly activated CACO-2 cells

**Figure 1:**
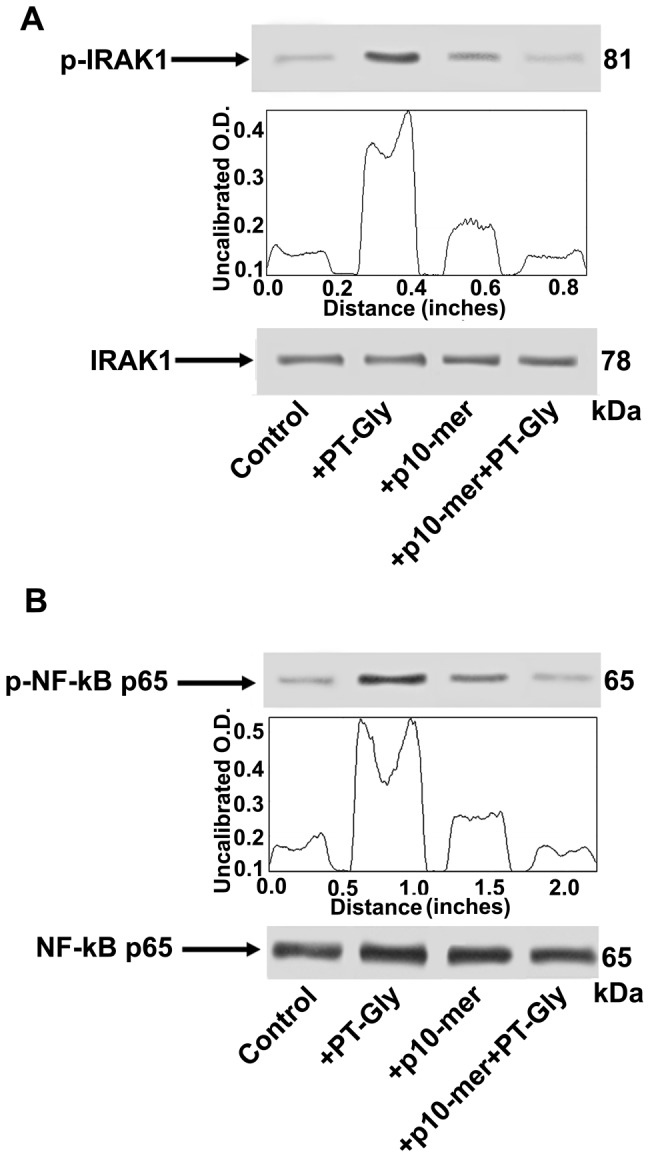
Inhibitory effect of gliadin peptide 10-mer on IRAK1 phosphorylation and NF-kB activation. CACO-2/TC7 cells, either unstimulated or stimulated with PT-Gly (1mg/ml), p10-mer (50 µg/ml), p10-mer (50 µg/ml) + PT-Gly (1mg/ml), were analyzed by Western blot for IRAK1 phosphorylation and NF-kB activation. (**A**) Phosphorylated levels of IRAK1 (p-IRAK1) were analyzed in whole cell extracts by Western blot with anti-phospho-IRAK1 antibodies; for control, the blotted membranes were stripped and reprobed with anti-IRAK-1 antibodies. Bound antibodies were visualized with HRP-conjugated IgG and immunoreactivity was assessed by ECL. (**B**) NF-kB activation was analyzed in whole cell extracts by Western blot with anti-phospho-NF-kB p65 Ser antibodies; for control, the blotted membranes were stripped and reprobed with anti-NF-kB p65 antibodies. Bound antibodies were visualized with HRP-conjugated IgG and immunoreactivity was assessed by ECL. Densitometric analysis was performed using ImageJ version 1.46 software and peaks were reproduced by reading the Western Blot bands. One example representative of 3 experiments.

Since CD is an autoimmune permanent disease supported by an inflammatory environment [4], in this study we analyzed the putative gliadin-dependent signaling pattern leading to inflammation. Cell lysates from CACO-2 cells, either unstimulated or stimulated with PT-Gly, p10-mer, p10-mer+PT-Gly, were analyzed by Western blot for IRAK1 phosphorylation. As expected, the results in Fig. 1A showed that PT-Gly induced IRAK1 phosphorylation, as revealed by anti-phospho-IRAK1 reactivity. When the cells were stimulated with PT-Gly (45 min) after pre-treatment with p10mer, IRAK1 phosphorylation appeared reduced.

With the aim to verify IRAK1 activation pathway, that is known to lead to the activation of NF-kB [12], we investigated the effects of p10-mer on PT-Gly induced p65 NF-kB activation. As expected, Western blot analysis of cell lysates revealed that PT-Gly stimulation (for 45 min) induced p65 activation, as revealed by anti-phospho-NF-kB p65 Ser reactivity (Fig. 1B). On the contrary, cells stimulated with PT-Gly after pre-treatment with p10-mer revealed phospho-NF-kB p65 levels virtually comparable to control cells. Cell lysates obtained using non IRAK1 and non NF-kB p65-specific IgG yielded no reactivity (data not shown).

**Figure 2:**
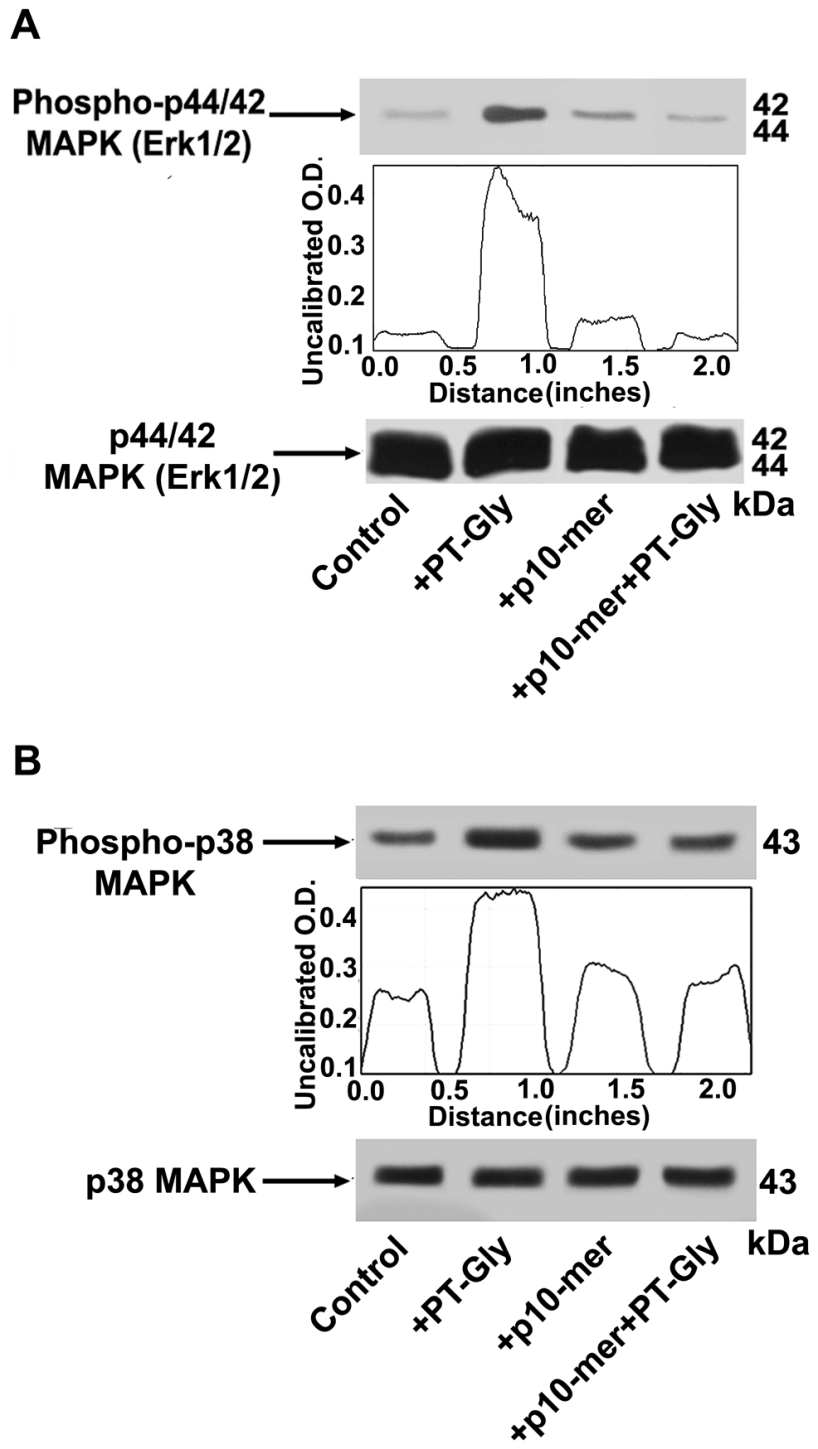
Inhibitory effect of gliadin peptide 10-mer on ERK and p38 MAPK phosphorylation. CACO-2/TC7 cells either unstimulated or stimulated with PT-Gly (1mg/ml), p10-mer (50 µg/ml), p10-mer (50 µg/ml) + PT-Gly (1mg/ml), were analyzed by Western blot for ERK and p38 phosphorylation. Phosphorylated levels of ERK were analyzed in whole cell extracts by Western blot with anti-phospho-ERK1/2 antibodies; for control, the blotted membranes were stripped and reprobed with anti-ERK1/2 antibodies. Bound antibodies were visualized with HRP-conjugated IgG and immunoreactivity was assessed by ECL. (**B**) Phosphorylated levels of p38 MAPK were analyzed in whole cell extracts by Western blot with anti-phospho-p38 MAPK antibodies; for control, the blotted membranes were stripped and reprobed with anti-p38 MAPK antibodies. Bound antibodies were visualized with HRP-conjugated IgG and immunoreactivity was assessed by ECL. Densitometric analysis was performed using ImageJ version 1.46 software and peaks were reproduced by reading the Western Blot bands. One example representative of 3 experiments.

The same cell lysates tested for IRAK1phosphorylation and p65 NF-kB activation were also analyzed by anti-phospho-ERK1/2 and anti-phospho-p38 antibodies. As shown in Fig. 2, ERK (Fig. 2A) and p38 MAPK (Fig. 2B) phosphorylation was evident in cell lysates from CACO-2 cells treated with PT-Gly (for 10 min); as shown, the same effector did not induce ERK and p38 MAPK phosphorylation after pre-treatment with p10-mer.

### Gliadin peptide 10-mer modulates COX-2 expression and activity in PT-Gly-stimulated CACO-2 cells

**Figure 3:**
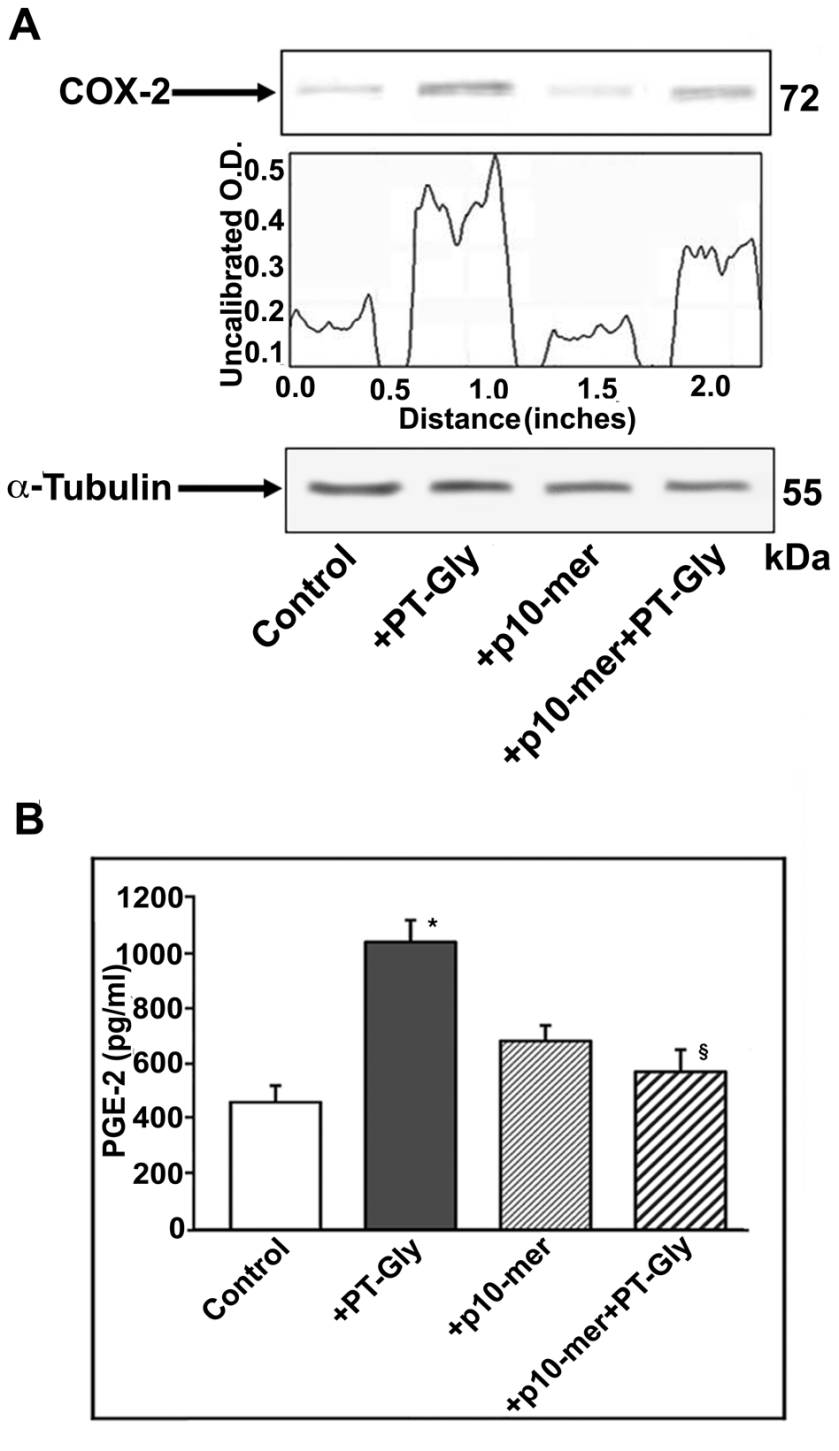
Regulatory effect of gliadin peptide 10-mer on COX-2 expression and activity in PT-Gly-stimulated CACO-2/TC7 cells. (**A**) CACO-2/TC7 cells, either unstimulated or stimulated with PT-Gly (1mg/ml), p10-mer (50 µg/ml), p10-mer (50 µg/ml) + PT-Gly (1mg/ml), were analyzed by Western blot for COX-2 expression. COX-2 expression was analyzed in whole cell extracts by Western blot with anti-COX-2 antibodies; for control, the blotted membranes were stripped and reprobed with anti-α tubulin antibodies. Bound antibodies were visualized with HRP-conjugated IgG and immunoreactivity was assessed by ECL. Densitometric analysis was performed using ImageJ version 1.46 software, peaks were reproduced by reading the Western Blot bands. (**B**) CACO-2/TC7 cells, either unstimulated or stimulated with PT-Gly (1mg/ml), p10-mer (50 µg/ml), p10-mer (50 µg/ml) + PT-Gly (1mg/ml) were analyzed for COX-2 enzymatic activity by measuring PGE-2 release with PGE-2 assay kit. Statistical analysis: *P<0.01 *versus* control; §P<0.05 *versus* PT-Gly. Results are expressed as mean ± SD; n = 3.

Since pro-inflammatory stimuli, often converging to the activation of ERK1/2 [18], promote COX-2 regulation, we preliminary investigated COX-2 expression in PT-Gly-treated cells. CACO-2 cells were pre-treated with p10-mer before PT-Gly treatment (for 3 hours). In Fig. 3A Western blot and densitometric analyses show that, when cells were pre-treated with p10mer, a partial inhibition of COX-2 expression occurred.

Since COX-2 can be regulated at transcriptional as well as post-transcriptional level, we analyzed the modulation of COX-2 enzymatic activity by measuring PGE-2 release in CACO-2 cells pre-treated with p10-mer and then stimulated with PT-Gly for 24 hours. p10-mer significantly inhibited PGE-2 production (P<0.05) (Fig. 3B).

### Effect of gliadin peptide 10-mer on cytokines production by PT-Gly activated CACO-2 cells

**Figure 4:**
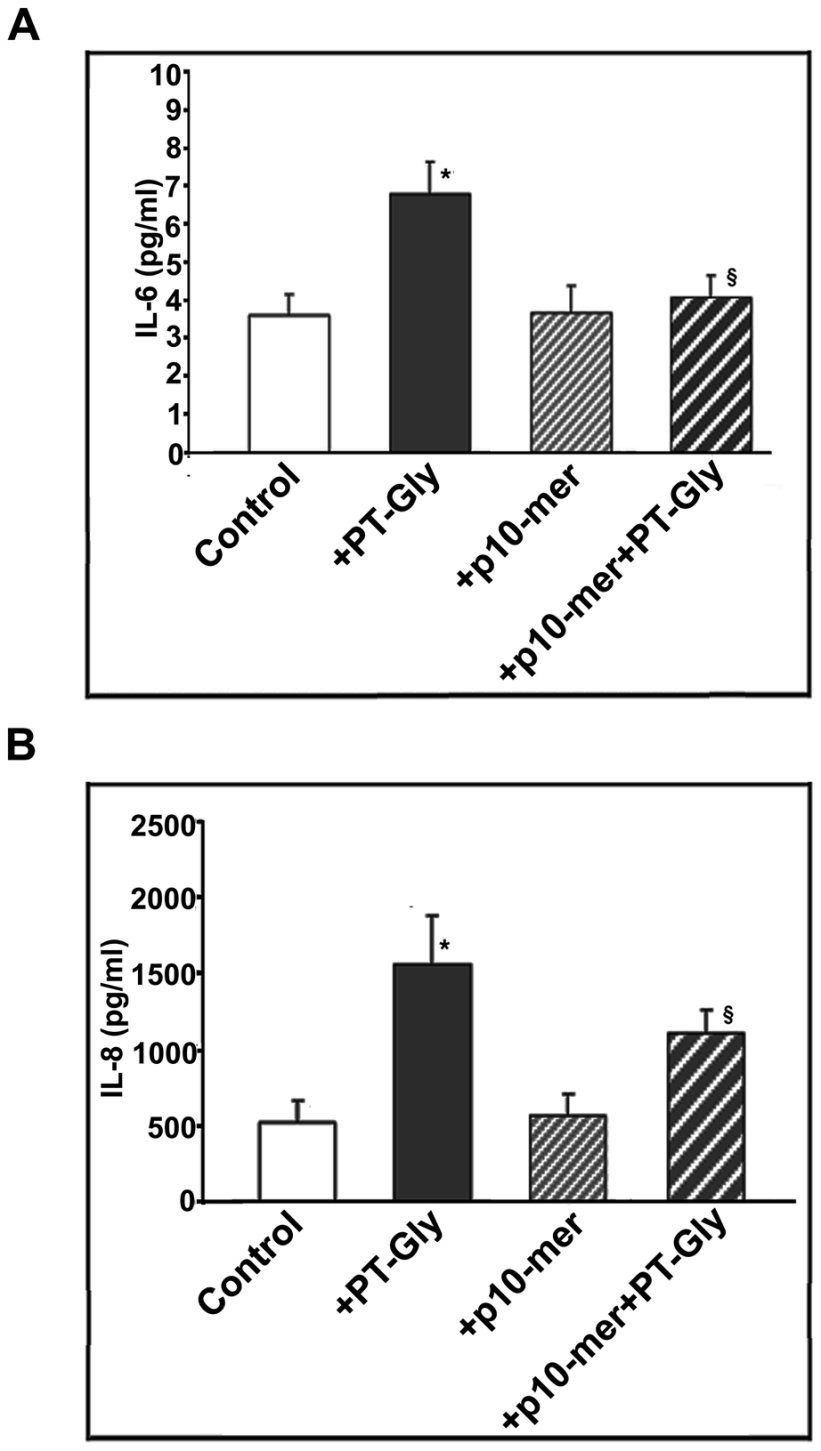
Inhibitory effect of gliadin peptide 10-mer on cytokine production. CACO-2/TC7 cells either unstimulated or stimulated with PT-Gly (1mg/ml), p10-mer (50 µg/ml), p10-mer (50 µg/ml) + PT-Gly (1mg/ml) were analyzed for cytokine production. Cell supernatants were analyzed for pro-inflammatory cytokines IL-6 (**A**) and IL-8 (**B**) release by an ELISA kit. Statistical analysis: *P<0.01 *versus* control; §P<0.05 *versus* PT-Gly. Results are expressed as mean ± SD; n = 3.

Since activation of signal transduction molecules, such as IRAK, NF-kB and MAPK is known to elicit cytokine gene expression, we analyzed IL-6 and IL-8 production in CACO-2 cells after PT-Gly stimulation for 24 hours, in the presence or in the absence of pre-treatment with p10-mer. As previously reported [19], PT-Gly stimulation induced both IL-6 and IL-8 secretion (Fig. 4). When cells were pre-treated for 1 hour with p10-mer, a significant modulation of IL-6 and IL-8 secretion was evident (P<0.05).

Since the gene of IL-17 was found to be expressed in CACO-2 cells and up regulated in some cases [20], we also tested IL-17 levels after PT-Gly stimulation for 24 hours, in the presence or in the absence of pre-treatment with p10-mer. Our results revealed that IL-17 levels were not detectable in both intracellular and supernatant assays (not shown). However, we cannot exclude that the expression levels of IL-17 were below the detection limit of the methods.

### Effect of gliadin peptide 10-mer on p31-43 activity and entrance in CACO-2 cells

**Figure 5:**
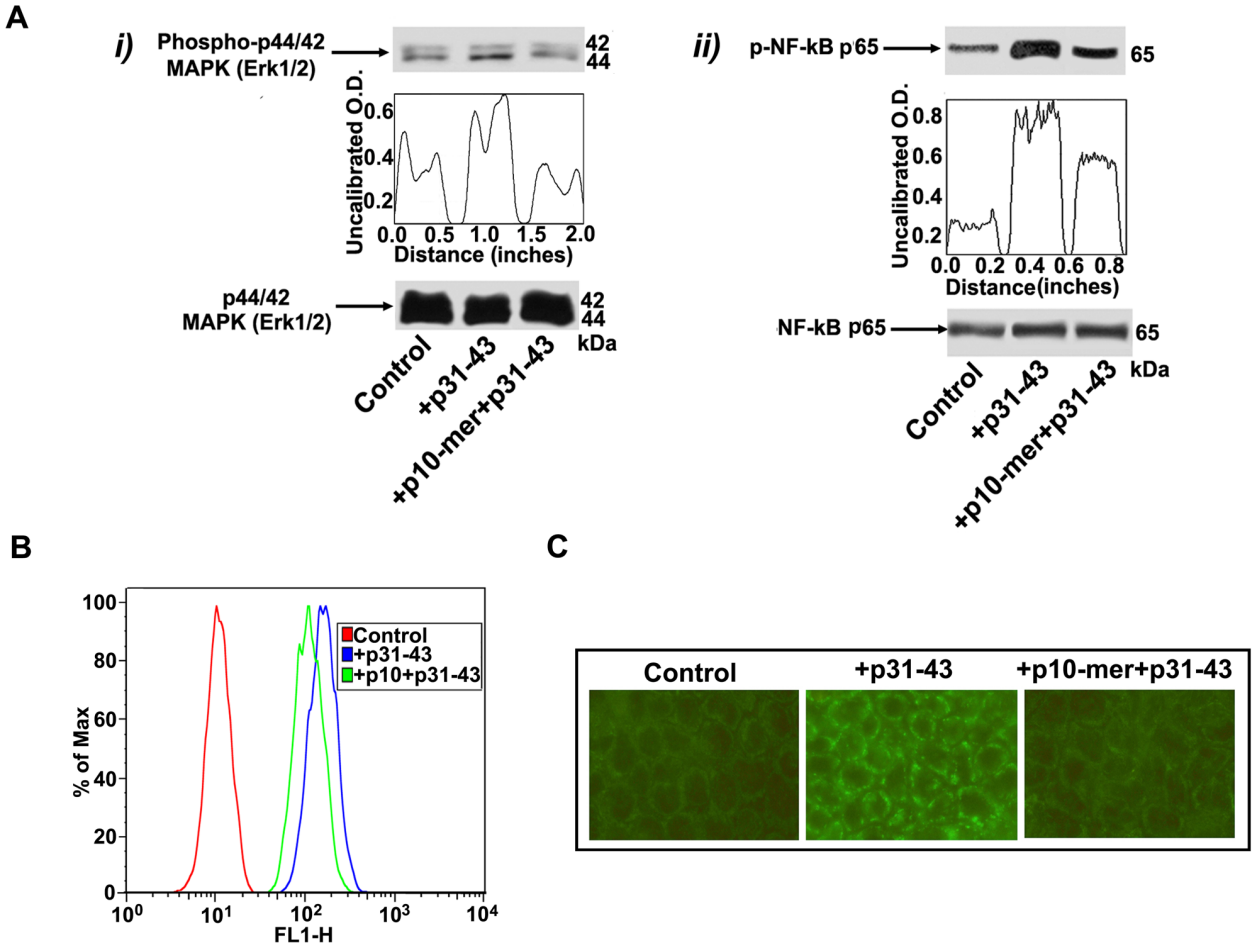
Effect of gliadin peptide 10-mer on p31–43 activity and entrance in CACO-2 cells. (**A**) CACO-2/TC7 cells, either unstimulated or stimulated with p31–43 (50 µg/ml), p10-mer (50 µg/ml) + p31–43 (50 µg/ml), were analyzed by Western blot for ERK phosphorylation and NF-kB activation. (**i**) Phosphorylated levels of ERK were analyzed in whole cell extracts by Western blot with anti-phospho-ERK1/2 antibodies; for control, the blotted membranes were stripped and reprobed with anti-ERK1/2 antibodies. Bound antibodies were visualized with HRP-conjugated IgG and immunoreactivity was assessed by ECL. (**ii**) NF-kB activation was analyzed in whole cell extracts by Western blot with anti-phospho-NF-kB p65 Ser antibodies; for control, the blotted membranes were stripped and reprobed with anti-NF-kB p65 antibodies. Bound antibodies were visualized with HRP-conjugated IgG and immunoreactivity was assessed by ECL. Densitometric analysis was performed using ImageJ version 1.46 software, peaks were reproduced by reading the Western Blot bands. One example representative of 3 experiments. (**B**) CACO-2/TC7 cells, either unstimulated or stimulated with biotinylated p31–43 (50 µg/ml), p10-mer (50 µg/ml) + biotinylated p31–43 (50 µg/ml), were immunostained with streptavidin-AlexaFluor and analyzed by a BD FACSCalibur flow cytometer. (**C**) CACO-2/TC7 cells, either unstimulated or stimulated with biotinylated p31–43 (50 µg/ml), p10-mer (50 µg/ml) + biotinylated p31–43 (50 µg/ml), were immunostained with streptavidin-AlexaFluor. The images were acquired using an Olympus U RFL fluorescence microscope.

Since it is important to know if the peptide p10-mer is an inhibitor of the entrance of the gliadin peptides in the cell, we preliminary investigated the effects of p10-mer on p31-43 induced p65 NF-kB activation. As expected, Western blot analysis of cell lysates revealed that p31-43 stimulation (for 45 min) induced NF-kB p65 phosphorylation. On the contrary, cells stimulated with p31–43 after pre-treatment with p10-mer revealed phospho-NF-kB p65 levels virtually comparable to control cells (Fig. 5A, i).

Similar findings were found on ERK phosphorylation. As shown in Fig. 5B, ERK phosphorylation was evident in cell lysates from CACO-2 cells treated with p31–43 (for 10 min); after pre-treatment with p10-mer, ERK phosphorylation was reduced, as revealed by densitometric analysis (Fig. 5A, ii).

Then, the entrance in the cell of the p31–43 was evaluated in the presence or in the absence of pre-treatment with the p10-mer. Our results strongly suggest that the p10-mer decapeptide is an inhibitor of the gliadin peptide entrance in the cell, as revealed by both flow cytometry (Fig. 5B) and immunofluorescence analysis (Fig. 5C).

## Discussion

It has been reported that gliadin sequence contains at least two immunological triggers, the antigenic epitopes (p33mer) and the danger signal, which leads to the activation of the innate immune system (p31–43), the so called “double soul of gliadin” [21]. The induction of an epithelial pro-inflammatory phenotype may alter the first mucosal defence against ‘toxic’ agents, leading to a wide perturbation of the regulatory mechanisms at the mucosal surface. The epithelial uptake of some toxic gliadin peptides, in particular p31–43, induces an intracellular pro-oxidative environment, favouring TG2 activation and leading to the innate immune response [10]. The inhibition of activation of the innate immune response in celiac disease might therefore be a useful therapeutic strategy to control disease evolution.

In this study, we investigated the ability to modulate the epithelial intestinal early response in celiac disease by a decapeptide (p10-mer, QQPQDAVQPF), already described to prevent activation of celiac peripheral lymphocytes by gliadin peptides and to down-regulate the adaptive immune response towards wheat prolamins [15].

Our results indicated first that this peptide is able to prevent IRAK1 phosphorylation and the consequent NF-kB activation, both induced by gliadin derived peptides, in CACO-2 cells. Moreover, when PT-Gly stimulated cells were pre-treated with p10-mer, a partial inhibition of both COX-2 protein expression and activation occurred.

It has been recently demonstrated that gliadin markedly activates immune cells, inducing the production of pro-inflammatory cytokines in all individuals, regardless of the clinical condition [22]. As expected [19], PT-Gly stimulation in CACO-2 cells induced secretion of two key pro-inflammatory cytokines, such as IL-6 and IL-8. In this regard, it is known that IL-8 is produced by stressed CACO-2 cells or gliadin-activated dendritic cells and it results overexpressed in response to dietary gluten [23]. IL-8 leads to a perturbation of the intracellular oxidative status in intestinal epithelial cells that reduces the production of PPAR-γ and elicits the NF-kB pathway activation [24]. Both these events play a key role in starting the inflammation in celiac epithelium. When cells were pre-treated for 1 hour with p10-mer peptide, a down-modulation of IL-6 and IL-8 secretion was evident.

The present study, using an intestinal epithelial cell line (CACO-2/TC7 cells), stimulated with the PT-Gly, also shows that pre-treatment with p10-mer peptide inhibited signal transduction pathway involving the IRAK1 kinase, resulting in inhibition of the activation cascade from the cytoplasm to the nucleus. In particular, there was a reduced activation of transcription factors, such as NF-kB, resulting in inhibition or reduced production of inflammatory mediators [3].

Moreover, p10-mer is also able to prevent COX-2 enzymatic activity, with consequent PGE-2 release, induced by PT-Gly. Increased levels of PGE-2 were described in the mucosa of celiac patients [25]. In particular, PGE-2 has been described to increase the intestinal paracellular permeability [26]. COX-2, which may be regulated by TLR/Myd88 dependent signals, as well as by Th1 cytokines, is known to promote inflammation via prostaglandins, including PGE-2. However, TNF-α-induced COX-2 expression and activity depend heavily on activation of ERK 1/2 and NF-kB [18]. Interestingly, in the present study we demonstrated that also ERK 1/2 phosphorylation induced by gliadin derived peptides was prevented by pre-treatment with p10-mer, further supporting the hypothesis that this peptide exerts an inhibitory effect on inflammatory response in CACO-2 cells.

In order to clarify the mechanism by which p10-mer is able to prevent the proinflammatory activity of gliadin peptide, it would be important to know if p10-mer may act as inhibitor of the entrance of the gliadin peptide in the cell. In this regard, the entrance in the cell of the 31–43 α-gliadin peptide was evaluated in the presence or in the absence of pre-treatment with the p10-mer. Our results strongly suggest that the p10mer decapeptide is an inhibitor of the entrance of the gliadin peptide in the cell.

Our findings on the effects of p10-mer on proinflammatory effects of gliadin peptides are consistent with previous observations by some of these authors, suggesting that p10-mer, and its naturally occurring–related peptide pRPQ, prevents some gliadin-dependent inflammatory pathways in intestinal epithelial cells and small bowel mucosa [27].

So far, the only known treatment for CD is the life-long exclusion of gluten-containing food from the diet or, alternatively, modifying the gluten sequences that act as epitopes in CD to abolish their immunogenic ability. Many approaches have been attempted to decrease the noxious effect of gluten in CD patients, such as degradation of gluten by exogenous enzymes [28], complexing gliadins in the gut by the use of polymeric binders diminishing the formation of immunogenic peptide [29], “sealing” tight junctions by the use of modulator of paracellular permeability to prevent the opening of tight junctions and restrict the passage of gluten peptides [30], interfering with immune recognition by the inhibition of tissue TG2 [31] or blockage of human leukocyte antigen (HLA) molecules [32] modulation of immune responses by peptide vaccination [33] and interference with inflammatory cell recruitment [34]. An alternative therapeutic option may rely on the reduction of the proinflammatory response induced by gliadin-derived molecules.

It has been already published [8] that inhibition of MAP kinase pathways can also be an effective approach in controlling the pathogenic cascade of celiac disease, showing that within the gliadin sequence there are at least two immunological triggers: the antigenic one, and the danger signal which leads to the activation of the innate immune system. This hypothesis may suggest that the inhibition of activation of the innate immune system in celiac disease might therefore be a useful therapeutic strategy to control disease evolution.

p10-mer has been described in the gluten sequence of a cereal cultivar only and the D and A residues, which confer the immunomodulatory activity to this peptide (13, 15), are very rare in the published gliadin sequences. However, we should not neglect the possibility that the sequence of p10-mer could be introgressed in a toxic cereal or administrated orally as drug to allow CD patients to consume gluten.

The oral delivery of preparations for treatment of CD is a matter of concern, since celiac inflammation starts in duodenal mucosa and a putative drug for this condition should be resistant to gastro-intestinal (GI) digestion to arrive unspoiled to the small intestine. p10-mer has been firstly identified in the peptic-tryptic digest of gluten (13). The gluten PT digestion performed *in vitro* mimics the proteolysis by human gastro-intestinal enzymes, therefore this finding demonstrates that p10-mer is resistant to the digestion by human GI system.

The results of the present study show that p10-mer peptide, can modulate the inflammatory response "in vitro", an important step to move towards new therapeutic strategies, based on the identification of "new targets" for a molecular innovative therapeutic approach. Turning off the inflammatory response, mediated by NF-kB, may in fact represent a key target in the immunotherapy of celiac disease.
